# Biologically Based Methods for Pest Management in Agriculture under Changing Climates: Challenges and Future Directions 

**DOI:** 10.3390/insects3041171

**Published:** 2012-11-09

**Authors:** Frank Chidawanyika, Pride Mudavanhu, Casper Nyamukondiwa

**Affiliations:** 1 Global Change and Sustainability Research Institute, School of Animal, Plant and Environmental Sciences, Faculty of Science, University of the Witwatersrand, Private Bag 3, Wits 2050, Johannesburg, South Africa; E-Mail: fchidawanyika@icipe.org; 2 Department of Conservation Ecology and Entomology, Faculty of AgriSciences, Stellenbosch University, Private Bag X1, Matieland 7602, South Africa; E-Mail: mudavanhu@sun.ac.za; 3 Department of Earth and Environmental Sciences, Faculty of Science, Botswana International University of Science and Technology (BIUST). Private Bag BO 041 Bontleng, Gaborone, Botswana

**Keywords:** climate change, integrated pest management, insect population dynamics

## Abstract

The current changes in global climatic regimes present a significant societal challenge, affecting in all likelihood insect physiology, biochemistry, biogeography and population dynamics. With the increasing resistance of many insect pest species to chemical insecticides and an increasing organic food market, pest control strategies are slowly shifting towards more sustainable, ecologically sound and economically viable options. Biologically based pest management strategies present such opportunities through predation or parasitism of pests and plant direct or indirect defense mechanisms that can all be important components of sustainable integrated pest management programs. Inevitably, the efficacy of biological control systems is highly dependent on natural enemy-prey interactions, which will likely be modified by changing climates. Therefore, knowledge of how insect pests and their natural enemies respond to climate variation is of fundamental importance in understanding biological insect pest management under global climate change. Here, we discuss biological control, its challenges under climate change scenarios and how increased global temperatures will require adaptive management strategies to cope with changing status of insects and their natural enemies.

## 1. Introduction

Anthropogenic climate change has the potential to significantly influence the biology of all organisms, but particularly ectotherms [1,2,3]; hence the evidence for a shift in ectotherm distribution in response to climate change [4,5]. While climate change is often associated with global mean annual temperature increases [6], likely favoring winter survival of insect pests, it is also coupled with increasing frequency and severity of extreme temperatures [7,8,9,10,11,12] that may modify predictions of insect population dynamics [13,14,15]. Short-term thermal fluctuations can be particularly stressful to small insects because their body temperatures are typically in equilibrium with ambient temperatures [16,17,18]. Hence, insects must be able to cope physiologically or compensate behaviorally with such changes in ambient temperature, both on spatial and temporal scales [15,19,20]. Physiologically, insects can adjust thermal tolerance over the short term, a phenomenon typically termed ‘hardening’ [21,22]. Over the longer term, thermal tolerance may be altered through acclimation in the laboratory or acclimatization in the field, and generally is a response to changes in environmental mean temperatures with e.g., season [23,24]. Hardening typically yields reversible physiological changes while acclimation and acclimatization can produce either reversible or irreversible physiological changes depending on the trait or whether developmental or maternal stages were exposed to temperature variation [22,24,25,26]. Martinat (1987) emphasized the importance of incorporating short-term undesirable weather transients (e.g., cold spells) in determining insect population dynamics [27]. Nevertheless, previous studies suggest considerable complexity when attempting to predict effects of transient weather patterns on insects’ physiology or life-history traits [28,29,30].

Biological control is a method of controlling pests that relies on predation, parasitism, herbivory and other natural mechanisms and can be an important component of integrated pest management (IPM) programs [31]. However, the efficacy of biological control using natural enemies depends on a complex but delicate relationship between natural enemies and their insect pest hosts whose balance can be offset by a changing climate. Environmental factors (e.g., temperature) directly affects the survival, development, reproduction and dispersal of pest insects and thus their potential biogeography and biotic potential [32,33]. It is well known that temperature fluctuations are the major factors affecting insect biology, activity and distribution of natural enemies in agro-ecosystems [34,35]. Moreover, several studies have indicated climate change affects several life history parameters e.g., generation time, fecundity, sex ratios and lifespan of parasitoids and their natural enemies [36,37]. Similarly, temperature extremes may reduce insect survival, reduce fecundity and retard natural enemies’ development [38].

Several studies have documented the likely effects of a changing climate on insect pest-natural enemies’ interactions [35,39,40,41]. Similarly, by assessing physiological traits of thermal tolerance and water balance, [42] it was shown that global climate change may affect the phenology of *Paractora dreuxi *Seguy (Diptera: Helcomyzidae). This review aims at examining the likely effects of climate change on insect biological control and how increased global temperatures will require adaptive management strategies to cope with changing status of insects and their natural enemies. We discuss how changes in global climate factors such as temperature increase will impact on [1] insect-natural enemy-host plant interactions, [2] insect/plant diversity [3] population growth/abundance [4] effectiveness of crop protection technologies, with special emphasis to biological control using natural enemies. This information has critical implications for sustainable pest management and food security, especially for developing countries in Africa, where food security is an urgent challenge [43].

## 2. Biologically-Based Pest Control Methods

Reducing public health and environmental risks associated with chemical pesticide use is of growing concern in developed and developing countries [44]. This has motivated the call for the adoption of biologically based IPM systems, an essential step towards reducing risks associated with the use of highly toxic pesticides [44]. 

Biological control can be classified into three basic categories namely conservation, classical and augmentation [45,46]. First, conservation biological control (CBC) involves the deliberate human practice aimed at promoting the survival and activity of natural enemies at the expense of pest populations [47]. For example, ecological strips consisting of selected non-crop plants can be deliberately created to provide food sources and overwintering shelters as well as protect local natural enemies from pesticide disturbances thereby enhancing CBC as successfully shown in cereals, cabbages, and fruit orchards [48,49]. 

Plant indirect defense mechanisms which rely on volatiles that call for natural enemies after pest damage, herbivore induced plant volatiles (HIPVs), have also been exploited to increase the activity of parasitoids in CBC systems [49,50]. In New Zealand and Australia the “attract and reward” is another form of the CBC approach where the use of HIPVs is combined with measures that increase key resources needed by natural enemies such as flowering plants in the commercial crop [50]. More recently, another form of CBC which combines stimulo-deterrent diversion tactics and conservation of parasitoids, through careful habitat manipulation has been shown to be effective against stem borers on sorghum *Sorghum bicolor* L. (Moench) and maize *Zea mays *L. [51]. Habitat manipulation has also been used to increase nectar availability, which increases the fecundity and longevity of some parasitoids [52,53,54], thereby increasing the efficacy of the program. For example, flowers are planted into commercial brassicaceous crops to enhance suppression of the diamondback moth, *Plutella xylostella* (L.) by its natural enemies [55].

Second, classical biological control (BC) involves collection of natural enemies from their area of origin and releasing them in the new area where their host was introduced accidentally [46,56]. This is of particular importance when the introduced pest species has no known alternative parasitoids indigenous to the area. However, the efficacy of a classical biological will depend on the newly released parasitoids to successfully establish populations that can compete in the new environment. For example in the USA, the alien yellow starthistle *Centaurea solstitialis* [L] has been successfuly controlled by insect natural enemies such as *Bangasternus orientalis* [Carpiomont], *Eusternopus villosus* (Boheman), *Urophora sirunaseva* (Hering) and *Chaetorellia succinea* (Costa) that attack the seed head [57].

Last, augmentative biological control (ABC) is the periodic release of large numbers of mass-reared natural enemies with the aim of supplementing natural enemy populations or flooding (*i.e*., inundating) pest populations with natural enemies [46,58]. It is commercially deployed in various cropping systems worldwide and two forms of ABC are distinguished namely the inundative approach and the seasonal inoculative method [46,59]. In the inundative release method, the BC agent is collected, mass-reared and released periodically in large numbers as for example a biotic insecticide to achieve immediate pest control in crops where viable breeding populations of the natural enemy are not possible [46]. This approach has been successfully applied in sugarcane for control of the sugarcane borer *Diatraea saccharalis* (F) in Latin America [60]. Other examples include the inundative release of common green lacewings *Chrysoperla carnea* (Stephens) to suppress *Erythroneura variabilis* (Beamer) and *E. elegantula* (Osborn) in vineyards [61]; and the release of *Trichogramma* spp for control of lepidopteran pests in vegetables, corn, rice, other cereals and cotton in Russia, China, SE Asia, Mexico and South America [62].

The seasonal inoculative approach differs from inundative method in that it is deployed in short-term crops, the production season of which is not longer than one year and where multiple pest generations occur [46]. The aim of the method is to obtain both immediate pest control as well as a build-up of the biological control agent population over the entire duration of the same production season [46]. Examples of the successful deployment of this technique include the release of *Trissolcus basalis * (Wollaston) for the control of *Nezara viridula* (L) in Brazil [63]; biological control of soybean stink bugs by inoculative releases of *T. basalis* [64]; control of the citrus blackfly, *Aleurocanthus woglumi* (Ashby) by inoculative releases of *Eretmocerus serius* (Silvestri) and *Amitus hesperidium* (Silvestri) in Cuba, Costa Rica, Mexico and Panama [46]; and the inoculation with *Metarhizium flavoviride * (Gams and Rozyspal) or *Verticillium lecanii * (Zimmerman) for the control of locusts *Schistocerca gregaria * (Forskal) [65] and grasshoppers [66].

## 3. Challenges in Biologically-Based Pest Management in Relation to Climate Change

Alterations in physiology and population dynamics, as a result of climate change, will bring new arrangements to levels of biological organization and ultimately ecological interactions in various species. In most geographic locations, shifts in climates may result in novel environmental conditions which are not only likely able to reduce the fitness but also deplete the quality and quantity of resources (e.g., food habitat) available for arthropod communities thereby threatening their existence in those areas. To counter this, arthropod populations facing unfavorable conditions may respond through either physiological or behavioral compensation [20,67], at both individual and population level, to better compete in the new environment; or they may migrate to new and favorable locations [68]. However, due to differences in the capacity to respond to various abiotic stressors and resource availability, fitness levels and dominance of various individual populations, they will be threatened, resulting in new species composition per locality, possibly dominated by the most adaptive ones [30]. Moreover, such modifications by living organisms, for better survival in life threatening environmental conditions, have already been hypothesized to pose new problems to them [69]. 

In turn, the resultant failure to successfully compete in the stressful conditions may lead to different species composition in both pests and their natural enemies. For agriculture, such changes are of importance as they create new structures in original pest abundance, emergence of formerly secondary to primary pests [32], colonization of new areas which were previously unfavorable [40,68] and more importantly the modifications of habitats [67,70,71,72], which may lead to reduction in numbers of natural enemies and parasitoids in the agro-ecosystem. Consequently, the changes may result in reduction in the efficacy of biological control due to alterations in the predator-prey relationships or lack thereof. In this section, an overview of challenges in biological control as a result of climate-induced changes in pest or parasitoid biology and general habitat modifications is discussed.

### 3.1. Habitat Fragmentation and Natural Enemy Diversity or Abundance

It is widely known that crop surroundings play a crucial role in the conservation of natural enemies and parasitoids [49,73,74,75]. However, in most agro-ecosystems, much attention is paid to the crop (e.g., planting, irrigation fertilization) as compared with the peripheral environment. Such an approach has not only ensured perpetual optimal crop growth, but dependable hosts for pests as well. Through elaborate efforts that ensure crop growth and survival, insect pest herbivory has been indirectly guaranteed. However, this is contrary to the micro environment faced by natural enemies dwelling in the periphery where the natural habitats may not be receiving similar attention resulting in their numbers being reduced. 

One consequence of changing climates may be habitat fragmentation of living organisms [1,76,77]. Thus, losses in suitable habitats will threaten the biodiversity and mere existence of organisms [78] including natural enemies or predators and parasitoids important for pest control in agro-ecosystems [75]. Consequently, the reduction or extinction of natural enemy populations will permit a pest build up, if unchecked, or over-reliance on alternative tactics for pest control which may be unsustainable, environmentally unfriendly and deplored by the consumers. 

### 3.2. Insect Biology and Physiology in Relation to Environmental Change

Temperature affects a range of biochemical and physiological processes and, along with water availability, is probably the major environmental factor affecting insect population dynamics at either the individual or population level [20,79,80]. At individual level, it has already been shown that factors such as temperature play a key role in determining insect fitness [81], field performance [82,83] and survival [84]. However, because of variability in response to thermal stress, for instance, which might be introduced by age, gender, ontogeny [85] and the species in question, mismatches in development and activity between pests and parasitoids may occur with cascading effects on the efficacy of biological control programs. Use of mass-reared parasitoids in augmentative efforts may be a challenge as well since the insects being introduced into novel environments, which might be stressful, may perform poorly [86]. Hence, in scenarios where pests perform better than their corresponding indigenous parasitoids under stress, the efficacy of a biological program will be dramatically reduced. However, some parasitoid species in the wild may benefit from rising winter temperatures. As discussed by Hance *et al*. (2007 and references therein), exposure to cold temperature in juvenile parasitoids results in reduced longevity of the adults [39]. Furthermore, cold exposure of adult parasitoids in the family Scelionidae reduces their longevity even after returning to optimal or warmer temperatures [39,87]. Low temperatures during development are also known to cause deformations and low fecundity in parasitoids. Basing on these limitations posed by low temperatures, a rise in winter temperatures may become beneficial to biologically-based pest management strategies for these specific parasitoid species, through improvements in their fecundity, development and longevity. At a population level, rising temperatures result in reduced generation time, rapid population growth and sometimes increased geographical ranges depending on resource availability [15,20]. However, such a positive correlation in temperature and population increase in pests is not uniform across different species even when facing similar conditions. This results in the asynchrony of life cycles of pests and parasitoids (or general reduction in populations of parasitoids required to effectively suppress pest populations. Such an asynchrony will create a temporal shortage or extinction of food resources for parasitoids whilst crop pest phenology is in line with the crop cycle. This consequently exerts pressure on the cropping system, especially in the cases of specialist parasitoids [88].

Furthermore, some pests may increase their invasion potential in relation to their ability to deal with changing climates either through phenotypic plasticity or variation in basal tolerance [89,90,91,92]. A classic example is the invasive maize stemborer *Chilo partellus* (Swinhoe), which was first introduced to Africa accidentally in Malawi but managed to establish itself in several African countries, becoming more destructive than the indigenous species in some instances [93]. Whilst the first introduction of *C. partellus* into Africa may have not been due to climate change, it has become apparent that its further establishment in several African regions follows a distinct pattern, which may be partly influenced by both climate and altitude [94]. Such pest dynamics increase pressure on the already-strained predator and parasitoid populations.

### 3.3. Chemical Ecology and Tritrophic Interactions in Agroecosystems

Climate induced changes in plant factors will affect quality and quantity of resources available for the insects resulting in variable direct and indirect consequences on the development times, size and fitness of both pests and parasitoids [95]. This may thus offset predation and parasitism [95], which sometimes reduces the efficacy of biological control programs. 

Perhaps another dimension of plant physiology which is likely to be modified, due to climate change, with a resultant impact on biological control is their secondary metabolism with a resultant impact on indirect defense mechanisms. It has already been shown that some plant species emit specific volatiles in response to elicitors in the saliva or secretions (during oviposition) of particular foraging herbivores, which call for natural enemies and parasitoids of the herbivores in question [96]. Other plant species, when attacked, have also been shown to emit volatile compounds which warn neighboring plants to prime their defense in advance in a phenomenon referred to as ‘eavesdropping’ [97]. Such volatiles have since been generally called herbivore induced plant volatiles (HIPVs) [97]. The HIPVs have been regarded as having evolutionary significance in that their production is only switched on when needed as opposed to constitutive mechanisms, which are always switched on. The latter may hence be ‘wasteful’ in terms of plant resource investment. However, recent studies have shown that plants undergoing abiotic stress respond by production of volatile isoprenoid compounds, perhaps, to avoid oxidative damage as a result of accumulation of reactive oxygen species in plants undergoing abiotic stress [98]. Isoprene compounds have since been shown to have the capacity to repel other specialist parasitoids, such as *Diadegma semiclausum *Hellen [99,100], and to influence herbivore feeding decisions [101]. It is therefore clear that isoprene production, as a result of abiotic stress, may influence plant-insect interactions in different agro-ecosystems. However, little is known of how the plants will prioritize their defense, in terms of volatile emission, when faced with biotic and multiple abiotic stressors associated with climate change. 

For biological control, changes in volatile composition important for defense may result in failure for parasitoids to locate their host as some may require specific volatile blends in order to perform the desired functions [102]. Moreover, it has been shown that environmental stressors such as temperature impact on fitness, olfactory perception [103] and ultimately the ability to of the insects to track their hosts [95]. Therefore, if such changes occur in the parasitoids, biological control programs using natural enemies will be rendered less effective.

### 3.4. Complexity in the Outcome of Climate Change Impacts on Natural Enemy Abundance and Population Dynamics

While it has increasingly become clear that the climate is changing [6], an accurate prediction of the consequent effects on species distribution remains a daunting task. In most ecosystems, baseline species distribution, before any change in climates, is determined by a host of interactive factors between abiotic and biotic factors of the species in question. However, bioclimatic models used to predict future species prediction have often omitted or failed to account for all of the important factors resulting in them being questionable [104,105]. In these models, behavioral, dispersal mechanisms and inter-specific interactions, which can be made by living organisms in changing climates, have often been neglected [106,107] or assumptions which bring uncertainties to the models have often been used [105]. Such lack of availability of reliable tools for prediction are a challenge to farmers, in particular, those who are currently or plan to use biological control as their main insect pest control tactic. Availability of reliable tools will not only boost the confidence in users of the model, but also avert catastrophes in pest management due to over-reliance on flawed models. 

Apart from bioclimatic models, resource constraints and challenges in experimental design have made empirical elucidation of the ecologically relevant behavioral and biological responses to climate change difficult. As a result, most inferences on the outcome of climate change are made from ecophysiological studies based on a single as opposed to multiple abiotic factors acting on different species. This is done despite increasing evidence of the differences in vulnerability or responses exhibited by some organisms when facing multiple as opposed to single stressors [108]. Possibly, through cross-tolerance [109] or additive effects of different stressors, such differences arise and may be species specific. It can therefore sometimes be misleading to rely on the inferences from the single-factor approach even though they serve as an important baseline indicator for individual physiological limits to stress tolerance. Such challenges bring new complications to farmers who are in principal, supposed to rely on accurate data and predictions in order to carry adequate planning and implementation of their crop protection strategies.

## 4. Future Directions

### 4.1. Environmental Stress Biology, Evolutionary Resilience and Ecologically Relevant Measures for Organism Response to Changes in Climates

Insect population dynamics can be strongly influenced by adaptive behaviors and traits [110]. Furthermore, insects/natural enemy extinction events, colonization rates and demographic rates are influenced by organisms’ adaptation in habitat selection, life history traits, niche breadth and dispersal behavior [111]. Recent studies suggest that in most cases, adaptive traits (e.g., thermal tolerance), significantly affect demographic dynamics and hence shape species distributions and population dynamics under a changing climate [86,112,113]. Similarly, in insects, low genetic variation in thermal adaptation can limit population growth and increase extinction risks in organisms living closer to their critical thermal limits [109,114]. However, although it is generally accepted that climate is changing, mitigating and coping with these effects remains an unresolved challenge [115,116]. In order to maintain or even improve biological control using natural enemies in a changing climate, several adaptive management strategies need to be implemented to cope with the changing status of insects and their natural enemies.

First and foremost, experimental protocols investigating the likely probable effects of climate change on insect-natural enemy interactions needs to be highly accurate. Although it has been documented that laboratory determined physiological traits (e.g., thermal tolerance) closely approximates their ability to cope with the stress under natural environments and are good indices of species fitness facing climate change [1], in most cases, there are ranging debates over the ecological relevance of experimental protocols used. For example, in thermal biology, previous studies have indicated that the methodological approach employed to determine an insect’s thermal tolerance can affect the types of insights that can be gained, and ecological relevance, of these thermal limits [20,117]. We therefore suggest that experimental protocols predicting climate change effects on insect population abundance should incorporate ecologically relevant measures of fitness traits (e.g., temperature tolerance) that are likely to occur in the species’ natural environment [118].

Moreover, forecasting efforts for insects’ responses to climate change has almost exclusively focused on the variation in mean temperatures [1,119]. However, it is expected that the magnitude and severity of temperature variances and extremes may also increase under future scenarios [7,12] with concomitant reduction in ectotherm fitness [113,120]. From a functional perspective, how changes in means and variances of temperature might affect the basal, phenotypic plasticity of temperature tolerance as well as life-history traits of ectotherms, remain poorly elucidated but are critical for predicting physiological responses in the wild [30,121]. We suggest that detailed analyses of changes in both means and variability of temperature for both pests and their natural enemies are a critical component of accurate forecasting of insect/natural enemy population-level responses to climate change. Furthermore, some other environmental stressors other than temperature may also impact synergistically or antagonistically on ectotherm fitness in a changing climate [118]. We therefore suggest that experimental protocols investigating ectotherm fitness in a changing climate should also incorporate other factors to evaluate possible interaction effects across factors on species’ fitness related responses [119]. In addition to interactive effects of physical factors, biotic factors such as parasitism or chemical ecology of various organisms in an interactive multitrophic system may be affected resulting in distortions in predator-prey relationships. In such a case, the fate of some species` populations in multitrophic systems will not be directly determined by the changes in climate on its population but by a species that is high or low in their food chain. Hence, wherever possible, experimental protocols that inform predictive models for changing climates should incorporate tests of other species, which might be of importance in the agro-ecosystems. 

The ability of organisms to mount physiological responses to variation in temperature at different time-scales may be an important component of insect/natural enemy persistence and thus efficacy of biological control under climate change scenarios [80,110,111]. From a theoretical standpoint, rapid evolution of thermal tolerance traits/phenotypes and their plasticity has been predicted [80,90]. Some studies indicated that if plasticity has an additive genetic basis, plasticity levels might evolve and likely contribute to evolutionary adaptation [90]. Phenotypic plasticity may buffer organisms upon introduction to novel and thermally unfavorable environments [92] and thus ensure survival when facing climatic stress [91]. However, current experimental protocols and crop protection specialists are ignoring the role of evolutionary processes in designing ways to protect and even improve biodiversity under global climate change [120,121,122]. We therefore propose that biological insect pest management programs using natural enemies should aim at developing resilient agro-ecosystems that maintain species’ and populations’ evolutionary potential [123,124]. Pörtner and Farrell (2008) point out that this may be possible through improvement in genetic diversity and processes that encourage continuous *in situ * evolutionary adaptation [125]. In agriculture, use of evolutionary processes to manage biotic interactions can be of importance as discussed in [126]. Through the help of artificial selections in crops, parasitoids or any other biological agents used to control may even be enhanced to gain competitive advantage over the pests intended for control when facing a selection pressure such as heat or drought. Such an approach is not new in agriculture as it has already succeeded in crop breeding, domestication of animals and pesticide resistance management [122,126]. Hence if given the investment priority in research, some pests may be managed even better under changing climates. 

Recent studies have proved that phenotypic plasticity can be used to enhance field performance of mass reared insects release for pest management [82,83]. We also argue that by manipulating thermal performance of mass-reared predators and parasitoids through acclimation, field performance in augmentative programs may at least be temporarily improved and hence improve pest management programs [86]. Similarly, [86] studies emphasize that pest management programs should incorporate valuable information acquired from studies of the evolutionary biology of thermal performance [82,83,127]. Likewise, this approach may be critical for improving efficacy of biological control programs in the face of climate change.

### 4.2. Monitoring, Ecological Assessments and Ecosystem Management

Apart from predictive models, regular monitoring and ecological assessments might be an important tool to accurately expose the impact of climate change on the distribution and abundance of pest and natural enemy populations. Whilst predictive models will be important in forecasting [128,129], their predictive power is often limited by assumptions, which may lack ecological considerations [104,130,131]. Hence ecological assessments and monitoring will generate huge ecological data sets that may be overlooked by physiological studies or predictive models. Such ecological factors are likely to give more valuable information of how agents of biological control will interact under changing climates. 

However, as the case with predictive models, assessments of various systems will require reliable ecological indicators in order to avoid making erroneous conclusions. These ecological indicators will serve as tools that portray the structure, function and composition of the ecosystems [132] or trends that will be happening over time [133] within and around the crops. Ecological indicators will therefore serve as early warning tools for detecting deficits in management strategies inflicted by climate change whilst also giving a database of various ecological transitions, which can be correlated to climate events. In turn, management strategies can be implemented earlier before extensive damage. However, as outlined by [132] use of ecological indicators can be problem if (i) the ecological indicators lack scientific integrity (ii) choice of indicators is confounded in management and (iii) the assessments are based on a small number of indicators. To counter this, concerted efforts between, scientists, farmers and policy makers should exist. Therefore, scientists should develop ecological assessment tools that are accurate but simplified to enable usage by a broad range of users. With adequate data collection from researchers, government agencies and farmers (at their local level) using well designed assessment tools, important conclusions can be drawn on the status of habitats, species composition and abundance in various regions.

Where parasitoid populations will be high but activity is reduced due to fragmented habitats, farmers can increase parasitoid attack on pests by use of companion cropping and plant indirect defense. Such is the case with the push-pull strategy used in the control of stemborers, which increases parasitisation of insect pests, by their parasitoids, through manipulation of the agro-ecosytem to lure parasitoids directly into the cropping system [50,51]. Augmentative strategies can also be used to boost parasitoid populations whilst using techniques such as acclimation to improve activity in variable environments [82,83,86]. Moreover, biological control can also be enhanced through feeding natural enemies with honey. This phenomenon has been shown to improve fecundity and longevity of hymenopteran parasitoids [52,53,54]. Hence identification for particular tactics and sometimes in combination will be important in tackling pests biologically under changing climates.

## 5. Conclusion

In conclusion, we propose that integrating physiology, population dynamics and climate mapping shows great promise for making robust predictions of the potential effects of global climate change on biodiversity [134]. Thus, to better elucidate the link between climate change, biodiversity and its impacts on biological control using natural enemies, a fruitful area of future studies would be in developing mechanistic physiological approaches (and ones that consider ecological factors) to understanding climate change effects on insect biology, biodiversity and population dynamics. Furthermore, there is significant evidence that species are evolving with climate change [135,136]. Hence future predictions of climate change effects on insect biodiversity should incorporate evolutionary potential [123,124,137].
